# Functionalized Fe_3_O_4_ Magnetic Nanoparticle Potentiometric Detection Strategy versus Classical Potentiometric Strategy for Determination of Chlorpheniramine Maleate and Pseudoephedrine HCl

**DOI:** 10.1155/2019/6947042

**Published:** 2019-02-25

**Authors:** Azza A. Moustafa, Maha A. Hegazy, Dalia Mohamed, Omnia Ali

**Affiliations:** ^1^Analytical Chemistry Department, Faculty of Pharmacy, Cairo University, Kasr-El Aini Street, 11562 Cairo, Egypt; ^2^Analytical Chemistry Department, Faculty of Pharmacy, October University for Modern Sciences and Arts (MSA), 11787 6th October City, Egypt; ^3^Analytical Chemistry Department, Faculty of Pharmacy, Helwan University, Ein Helwan, 11795 Cairo, Egypt

## Abstract

Nanosized adsorbents when used in potentiometric methods of analysis usually show better performance rather than the traditional potentiometric approach; this is attributed to the high specific surface area of the nanomaterial used in addition to the lack of internal diffusion resistance, thus improving their adsorption capacity. In the presented work, a rapid and sensitive potentiometric determination of chlorpheniramine maleate (CPM) and pseudoephedrine hydrochloride (PSE) in pure form, in pharmaceutical preparation, and in biological fluid was developed based on functionalized magnetic nanoparticles (Fe_3_O_4_). This strategy was compared with the classical potentiometric strategy. Three types of sensors were constructed using phosphotungstic acid (PTA), *β-*cyclodextrin (*β-*CD), and *β*-cyclodextrin-conjugated Fe_3_O_4_ magnetic nanoparticles for the potentiometric determination of each of CPM and PSE. The prepared sensors were characterized in regards to their composition, life duration, working pH range, and response time. The sensors have demonstrated promising selectivity to CPM and PSE in the presence of pharmaceutical formulation excipients, plasma matrix, and a diversity of both organic and inorganic interfering materials. The developed sensors have displayed good responses. Statistical comparison of the achieved results with a reported method has revealed no significant difference regarding both accuracy and precision.

## 1. Introduction

**Figure 1:**
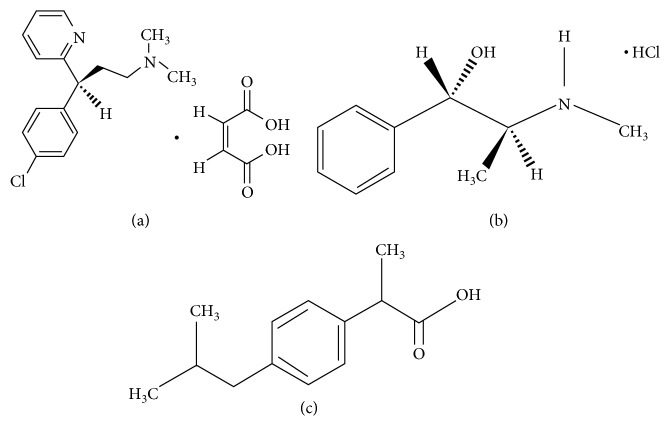
Chemical structure of (a) CPM, (b) PSE, and (c) IBF.

Chlorpheniramine maleate (CPM) is (3-(4-chlorophenyl)-*N*,*N*-dimethyl-3-pyridin-2-yl-propan-1-amine (Figure 1(a)). CPM is considered as a first-generation alkylamine antihistamine which is mainly prescribed to inhibit allergy symptoms as rhinitis and urticaria. CPM can give rise to a moderate degree of sedation [1]. Pseudoephedrine hydrochloride (PSE) is also known as [(+)-threo-*α*-[1-methylamino) ethyl] benzyl alcohol] hydrochloride (Figure 1(b)). PSE is a sympathomimetic amine drug which is administered for its bronchodilator and nasal decongestant effects as it has the ability to contract the swollen mucous membranes, decrease the nasal airway obstruction, and reduce tissue hyperemia [2]. Ibuprofen (IBF) is [(2S)-2-(4-isobutylphenyl) propanoic acid] (Figure 1(c)). IBF is a nonsteroidal anti-inflammatory drug (NSAID) which is utilized due to its ability to relief pain, treat fever, and reduce inflammation [3].

CPM, PSE, and IBF are coformulated drugs in a single dosage from which is utilized for treating the symptoms cough and common cold.

A detailed survey for the reported methods that were utilized for the analysis of CPM, PSE, and IBF has revealed that these drugs were determined either singly or in their binary mixtures by spectrophotometry [4, 5], HPTLC [6], HPLC-MS and GC-MS [7–9], HPLC-UV [10], capillary electrophoresis [11, 12], and potentiometry [13–22]. However, only one method was found for the simultaneous determination of the three drugs based on liquid chromatography with experimental design [23].

Analytical methods involving molecular host-guest approaches have attracted tremendous concern due to their high selectivity. Cyclodextrins as ionophoric polymers are extensively recognized to compose stable inclusion complexes with a variety of organic, inorganic, and biological guest molecules in their lipophilic cavities while demonstrating high molecular selectivity as well as enantioselectivity [24, 25].

Functionalized magnetite nanoparticles have the ability to be widely dispersed in the analyte solution and intensely stimulate the chemical reaction between their functional groups and the target analyte, and this is attributed to their considerably lower diffusion layer thickness in contrast to their bulk counterparts. Magnetic nanoparticles are among the particles that were actively examined, where based on their interesting magnetic properties, they are evolving as possibly useful tools for a wide variety of applications [26] due to, for example, their successful usage as a new approach in potentiometric analysis of drugs in different pharmaceutical preparations [27].

In view of the previously mentioned points of view, we aimed to develop a new potentiometric approach utilizing magnetic nanoparticles for the rapid and sensitive quantification of CPM and PSE in their bulk powder, pharmaceutical dosage form, and plasma samples without interference from IBF. The proposed study has demonstrated certain encouraging improvements to the use of *β*-cyclodextrin. The achieved results were compared to the classical potentiometric methods.

## 2. Experimental

### 2.1. Apparatus

Potential measurements were carried out using a Jenway digital ion analyzer model 3505 (Jenway, UK) with Ag/AgCl double junction reference electrode (Aldrich, USA). The pH adjustment was performed utilizing a Jenway pH glass electrode (Jenway, UK). Imaging of Iron oxide magnetite nanoparticles was performed with the aid of a JEOL JEM-2100 high-resolution transmission electron microscope where the accelerating voltage was adjusted to 200 kV.

### 2.2. Chemicals and Reagents

#### 2.2.1. Pure Samples

Chlorpheniramine maleate (CPM) with a purity of 99.72 ± 1.065 and pseudoephedrine HCl (PSE) with a purity of 99.45 ± 1.011 were provided from Egyptian International Pharmaceutical Industries Co. (EIPICO), Cairo, Egypt. Ibuprofen (IBF) with a purity of 100.05 ± 1.357 was provided from GlaxoWellcome, Egypt. The purity of the three drugs was checked by a reported method [23].

#### 2.2.2. Market Sample

Sinlerg® coated tablets (Eva Pharma for pharmaceuticals and medical appliance S.A.E, Haram, Giza, Egypt) with batch number: 212314 were obtained from the local market. Each tablet is composed of 2 mg of CPM, 30 mg of PSE, and 200 mg of IBF as labelled.

#### 2.2.3. Chemicals and Reagents

**Figure 2:**
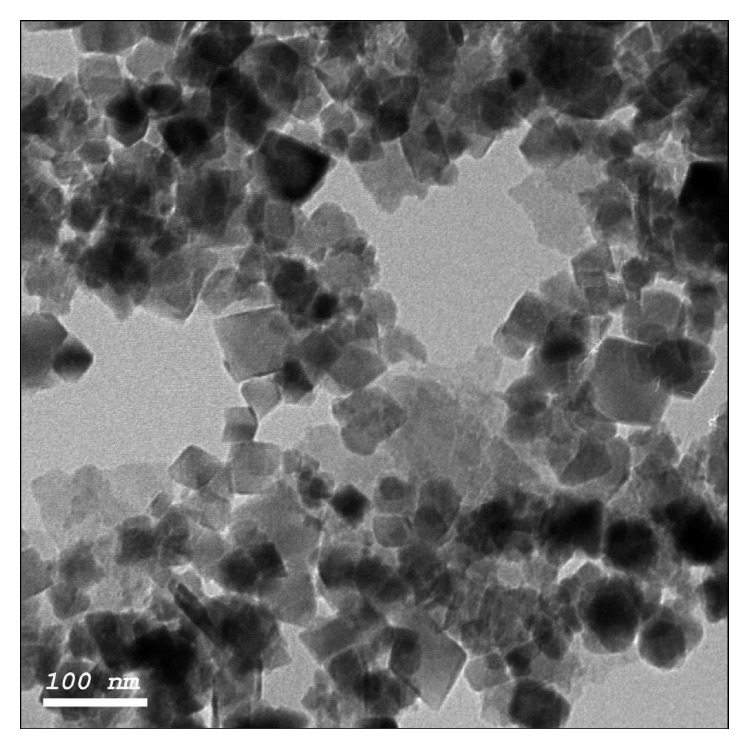
TEM micrograph of Fe_3_O_4_ nanoparticles.

All chemicals and reagents used were of analytical reagent grade. High-molecular weight polyvinyl chloride (PVC), *β*-cyclodextrin (*β*-CD), dibutyl phthalate (DBP), and phosphotungestic acid 99.97% (PTA) were purchased from Aldrich (Steinheim, Germany). Tetrahydrofuran (THF) was purchased from Merck (Darmstadt, Germany). Hydrochloric acid, sodium hydroxide, urea, potassium chloride, calcium chloride, and potassium dihydrogen phosphate all were purchased from El-Nasr pharmaceutical chemicals (Cairo, Egypt). Plasma was purchased from VACSERA (Egypt). Iron oxide (Fe_3_O_4_) magnetite nanoparticles (5 nm diameter) were purchased from Nanotech Egypt for photoelectronics Co. (6th of October City, Egypt). According to the manufacturer procedure, Fe_3_O_4_ was synthesized via the coprecipitation method. This method is based on alkaline coprecipitation of ferric and ferrous salts in aqueous solution [28, 29]. In addition, imaging was performed to confirm the size and shape as demonstrated in Figure 2.

### 2.3. Standard Solutions

#### 2.3.1. Stock Solutions

The stock solutions of both CPM and PSE were prepared separately in the concentration of (10^−2^ M) using distilled water as a solvent.

#### 2.3.2. Working Solutions

The working solutions of CPM (10^−8^–10^−3^ M) and PSE (10^−9^–10^−3^ M) were freshly prepared via dilution from the prepared stock solutions using distilled water.

Both the stock and working solutions were stable for at least one month when kept in a refrigerator at 4°C.

### 2.4. Procedures

#### 2.4.1. Precipitation of the Ion Exchanger

Two aliquots of 10 mL of (10^−2^ M) aqueous standard CPM and PSE solutions were transferred in two different beakers followed by the addition of 10 mL of aqueous (10^−2^ M) of PTA solution on each beaker. The two prepared solutions were mixed well for 5 minutes. The obtained precipitates were filtered through Whatman filter papers and then washed several times using cold distilled water till the precipitate is chloride-free as indicated when tested by AgNO_3_ solution. The precipitate is then dried at room temperature and ground to be in the form of fine powder. Elemental analysis (carbon, hydrogen, and nitrogen) was performed to check both the formation and purity of the ion-associates as well as the chemical composition of the precipitates as abridged in Table 1.

#### 2.4.2. Fabrication of PVC-Based Membrane Sensors (Sensors 1, 2, 4, and 5)

For the preparation of sensors 1 and 4, 10 mg of the ion exchangers (CPM-PT and PSE-PT) was separately mixed in glass Petri dishes (5 cm diameter) with 0.35 ml of DBP and 0.19 g PVC. While for the preparation of sensors 2 and 5, the following materials were thoroughly mixed with each other: 0.19 g PVC with 0.35 ml DBP and 0.04 g *β*-CD. Subsequently, the obtained mixtures were dissolved in 5 ml THF in Petri dishes (5 cm diameter) and homogenized carefully.

The Petri dishes were left overnight at room temperature after being covered with filter papers in order to permit for solvent evaporation. Master membranes were formed with a thickness of about 0.1 mm. Disks of about ≈10 mm diameter were cut from the master membranes, with the aid of a cork borer. The disks were then pasted utilizing THF to interchangeable PVC tips that were clipped into the end of the electrodes glass bodies. Equal separate volumes of (10^−2^ M) CPM or PSE and (10^−2^ M) KCl were mixed well where the obtained solution was considered to be the internal reference solution. However, the internal reference electrode was formed through the immersion of an Ag/AgCl wire (1 mm diameter) in the internal reference solution. Each formed electrode (sensor) was preconditioned by being dipped in (10^−2^ M) CPM and PSE solutions for 24 h.

The electrochemical cell for the potential measurements could be represented as follows: Ag/AgCl (internal reference electrode)/1.0 × 10^−2^ M CPM or PSE solutions, 1.0 × 10^−2^ M KCl (internal reference solution)//PVC membrane//test solution (pH 4–8) and (pH 4–7) for CPM and PSE, respectively//Ag/AgCl double junction reference electrode. The electrodes were usually kept in deionized water in between the measurements. The fabrications of the four ISE electrodes (sensors 1, 2, 4, and 5) are described in Table 2.

#### 2.4.3. Coating of Nanoparticles with *β*-CD and Fabrication of Functionalized Fe_3_O_4_ Nanoparticles Membrane Sensors (Sensors 3 and 6)

Coating of nanoparticles with *β*-CD, as ionophoric polymer, was achieved through the addition of 0.04 mg *β*-CD which is dissolved in 0.35 mL DBP as a plasticizer. The components were carefully mixed with 5 mL THF until the mixture is completely homogenous. Then, 1 mL of the nanoparticles solution was added, followed by sonication for 15 minutes, and finally, the mixture was left to allow for the evaporation of THF.

For the fabrication of the functionalized Fe_3_O_4_ nanoparticles membrane sensors, the same procedure for the preparation of sensors (2 and 5) was repeated as explained in Section 2.4.2.

However, in order to allow for the formation of the inclusion complex between the nanoparticles' functional groups and CPM and PSE, 0.1 mL of the final magnetic fluid was added to the inner solution (which is composed of equal volumes of (10^−2^ M) CPM or PSE and (10^−2^ M) KCl) of the membrane electrode of the prepared sensors. The internal reference electrode was formed through the immersion of a Ag/AgCl wire (1 mm diameter) in the internal reference solution. The designed sensors (3 and 6) were preconditioned by being dipped in (10^−2^ M) CPM and PSE solutions for 24 hours. The induced membrane potential was measured while applying a magnetic field with the aid of a magnetic stirrer, during the time of sample measurement [26]. The calibration curves were constructed by plotting *E* (mV) against the corresponding negative concentration logarithm of CPM or PSE.

#### 2.4.4. Sensors Calibration

The conditioned sensors were calibrated through their immersion in about 50 mL of the working standard solutions equivalent to 1 × 10^−8^–1 × 10^−3^ M and 1 × 10^−9^–1 × 10^−3^ M for CPM and PSE, respectively. The sensors were in conjunction with the Ag/AgCl reference electrode and were permitted to equilibrate with constant stirring utilizing a magnetic stirrer throughout the whole process. The sensors were usually washed with distilled water in between the measurements. The electrode potential of each sensor was recorded after being stabilized to ±2 mV and was plotted against the corresponding negative logarithmic concentration of CPM and PSE. Consequently, the constructed calibration curves were then utilized for the measurement of unknown samples.

#### 2.4.5. Effect of pH

As the developed potentials of the six fabricated sensors is influenced by the pH, the effect of pH was examined within the range of 2–10 utilizing the solutions of CPM and PSE in a concentration equivalent to 10^−4^ M and 10^−3^ M, respectively. Both dilute sodium hydroxide and hydrochloric acid solutions were used for pH adjustment to the required value. The acquired potential for each pH value was recorded.

#### 2.4.6. Sensors Selectivity

The interference which foreign ions might perform on the response of each fabricated sensor to its primary ion was evaluated using the potentiometric selectivity coefficient—log *K* ^pot.^ (primary ion, interferent). The selectivity coefficients were calculated through measurement of the developed potentials from 10^−3^ M aqueous of each of CPM and PSE solution and then for 10^−3^ M aqueous interferent solution. For calculating the potentiometric selectivity, the following equation was used:

(1) $$\begin{array}{c} \log\left(K^{\text{pot}.}\text{primary ion interferent}\right)=\left[\frac{E_{\text{D}}-E_{\text{M}}}{2.303RT/Z_{\text{D}}F}\right]+\left[1-\frac{Z_{\text{D}}}{Z_{\text{M}}}\right]\log\left[D\right], \end{array}$$

where *E*_D_ is the potential measured in 10^−3^ M of drug solution for CPM or PSE, *E*_M_ is the potential measured in 10^−3^ M interferent solution, *Z*_D_ and *Z*_M_ are the charges of CPM or PSE and interfering ion, respectively, and 2.303*RT*/*Z*_D_ *F* represents the slope of the investigated sensor (mV/concentration decade).

#### 2.4.7. Application to Pharmaceutical Dosage Forms

Ten Sinlerg® tablets were accurately weighted, powdered, and mixed well. The weight equivalent to one tablet was transferred into a 50 mL volumetric flask, and the volume was completed using distilled water. Then, further dilution with the same solvent was carried out to achieve a concentration of 10 and 150 *μ*g/mL of CPM and PSE, respectively (IBF is sparingly soluble in water). The fabricated sensors (3 and 6) were dipped in the solution and were kept under constant stirring using a magnetic stirrer throughout the whole process. The sensors were in conjunction with the Ag/AgCl reference electrode.

#### 2.4.8. Determination of CPM and PSE in Spiked Human Plasma

One milliliter of the working standard solutions of CPM and PSE equivalent to 10^−5^, 10^−6^, and 10^−7^ M was added individually into a set of stoppered tubes comprising 9 mL of human plasma, and then the tubes were shaken for 1 min. The fabricated sensors (3 and 6) were dipped in the plasma solutions and were kept under constant stirring using a magnetic stirrer throughout the whole process. The sensors were in conjunction with the Ag/AgCl reference electrode. The acquired potential values were measured, and the concentrations of CPM and PSE were calculated from the corresponding previously computed regression equations.

## 3. Results and Discussion

Magnetite Fe_3_O_4_ nanoparticle is currently one of the most widespread magnetic nanoparticles which are extensively utilized in several applications. Based on the specific properties of nanoparticles, including their extreme small size, large surface area to volume ratio, and non-existence of internal diffusion resistance, this has led to providing improved kinetics for adsorbing contaminants from aqueous solutions [30]. Besides, the insertion of *β*-CD on the surface of magnetic nanoparticles was expected to enhance both their stability and dispersion in aqueous solutions, thus leading to increase in their surface areas and consequently improving their adsorption capacities [31–33].

**Figure 3:**
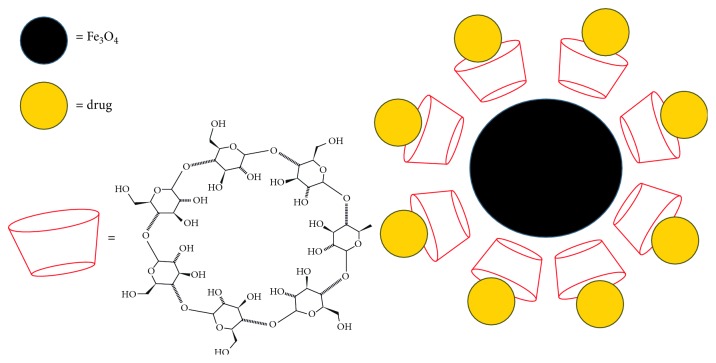
Schematic diagram representing the functionalization of Fe_3_O_4_ nanoparticles.

The magnetic nanoparticles utilized in the presented study were manufactured by a classical coprecipitation method using *β*-CD as ionophoric polymer [34]. Through the inclusion of the prepared magnetic fluids into the inner solution of the membrane electrode, this would allow for the prompt and symmetric dispersion of the ion exchanger-functionalized magnetic nanoparticles in the solution [26, 35]. When a magnetic field is applied, the magnetic nanoparticles will be aggregated to the inner side of the polymeric membrane while the adsorbed ionophore and plasticizer on the nanoparticles will dissolve on the surface of the membrane resulting in a substantial potentiometric response [26, 35] (Figure 3).

The introduced work is devoted to evaluate the possibility of quantitative analysis of CPM and PSE by using ion selective electrode (ISE) sensors by means of PTA as ion exchanger, *β*-CD that forms host-guest inclusion complexes, and *β*-CD-conjugated Fe_3_O_4_ magnetic nanoparticles. A comparative study was held between traditional potentiometric strategy and potentiometric detection strategy based on functionalized Fe_3_O_4_ magnetic nanoparticles showing their advantages. The performance characteristics of the fabricated sensors were assessed in accordance with the IUPAC recommendations [36].

### 3.1. Sensors Fabrication

Generally, the solubility product of PTA ionic exchangers is low and it has suitable grain size. CPM and PSE were found to react as a monovalent cation, and they formed 3 : 1 ion association complex with PTA which was confirmed both by elemental analysis (Table 1) as well as by the achieved Nernstian slopes.

### 3.2. Sensors Performance Characteristics and Response Time

**Figure 4:**
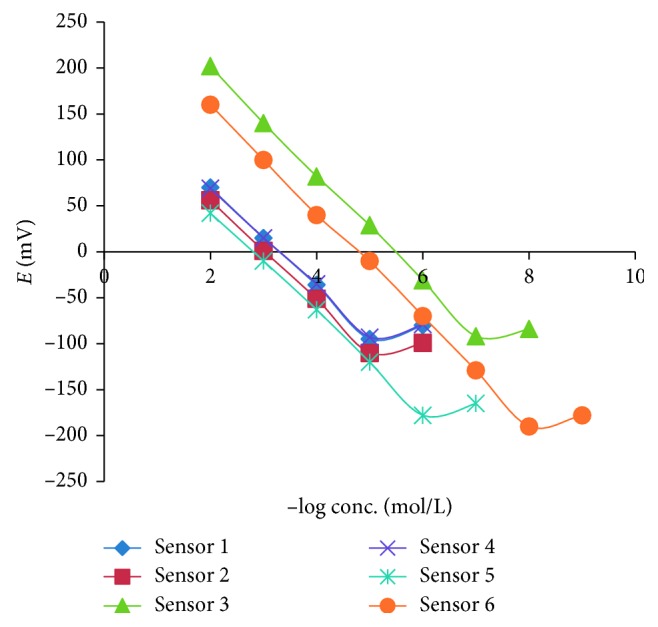
Profile of the potential in mV to −log concentration of CPM and PSE in mol/L obtained with the proposed sensors.

The electrochemical performance characteristics of the examined CPM and PSE sensors were assessed in accordance with the IUPAC recommendations [36] where the obtained results are abridged in Table 3. Typical calibration plots are demonstrated in Figure 4. As the electrodes responds mainly to the activity of the analytes (as cations) rather than their concentration, the slopes of the calibration plots have displayed a deviation from the ideal Nernstian slope (60 mV). The slopes of the calibration plots were 54.60, 55.10, 53.70, and 55.00 mV/concentration decade for sensors (1, 2, 4, and 5), respectively. However, Fe_3_O_4_ electrodes have exhibited the nearest value to the ideal Nernstian slope with the values of 58.17 and 57.79 mV/concentration for sensors (3 and 6), respectively. The investigated Fe_3_O_4_ electrodes have demonstrated constant potential values in between different measurements where the slopes of the calibration curves were not altered by more than ±2 mV/decade throughout the stability periods of the developed sensors. Additionally, the slopes were not changed significantly but revealed a gradual decrease in sensitivity.

The examined Fe_3_O_4_ electrodes have displayed a fast response time. The time desired by the electrodes to accomplish ±1 mV values of the equilibrium potential after increasing the analytes concentration to 10-folds was about 5–10 s.

### 3.3. Effect of pH

**Figure 5:**
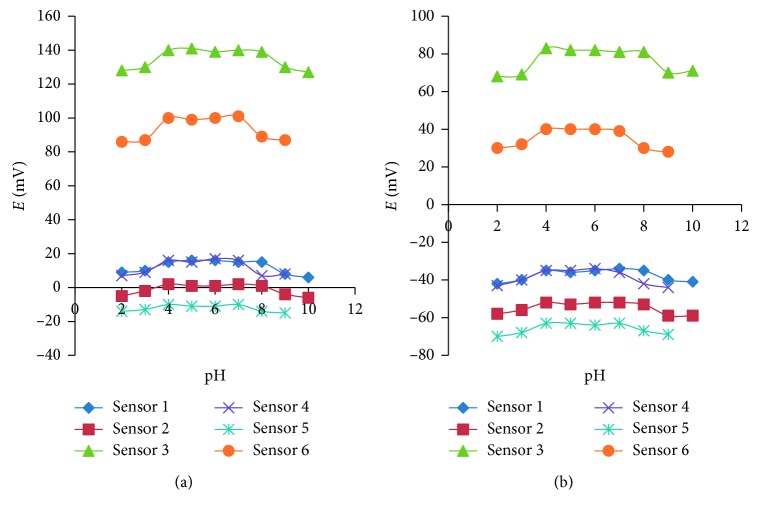
The effect of pH on the developed sensors using (a) 10^−3^ M and (b) 10^−4^ M of CPM and PSE solution.

In order to optimize the experimental conditions to allow for the quantitative analysis with the ion selective electrodes, the effect of pH on the response of the examined electrodes was investigated using 1 × 10^−4^ and 1 × 10^−3^ M solutions of CPM and PSE. The potential-pH profile for sensors (1, 2, and 3) has demonstrated that the responses were fairly constant within the pH range 4–8, whereas the responses of the sensors (4, 5, and 6) were constant within the pH range 4–7. The effect of pH on the responses of the developed electrodes is illustrated in Figure 5.

### 3.4. Sensors Selectivity

The potentiometric selectivity coefficients of the investigated sensors in the presence of coformulated drug (IBF), excipients present in the pharmaceutical formulations in addition to some inorganic cations such as K^+^, Na^+^, and Ca^2+^ which are normally present in biological fluids, are all demonstrated in Table 4. The obtained results have revealed that the Fe_3_O_4_ sensors (3 and 6) exhibited higher selectivity than the classical sensors (1, 2, 4, and 5) for the quantitative analysis of CPM and PSE.

The proposed method was validated according to ICH guidelines, and it was found to be linear within the ranges from 10^−8^ to 10^−2^ with correlation coefficient close to one. The method displayed good accuracy and precision, where the accuracy was found to be between 99.45 and 101.85 and precision was less than 2. The calculated LOD was found to be 5 × 10^−8^ for sensor 3 and 4 × 10^−9^ for sensor 6 which indicates the sensitivity of the proposed method. Due to these obtained promising results, the method was applied for the estimation of CPM and PSE both in pharmaceutical formulation and human plasma.

### 3.5. Potentiometric Determination of CPM and PSE in Pharmaceutical Formulation

Due to the higher selectivity of the Fe_3_O_4_ sensors (3 and 6), they were applied for the quantitative determination of CPM and PSE in tablet dosage form, where the recovery% ± S.D. was found to be 102.26 ± 1.204 and 101.09 ± 1.106 for CPM and PSE, respectively. The obtained results were compared by using the reported method and verified the ability of the proposed sensors to be applied for the potentiometric estimation of the target drugs with no interference from IBF as a coformulated drug or from the commonly used excipients (Table 5).

### 3.6. Potentiometric Determination of CPM and PSE in Spiked Human Plasma

Fe_3_O_4_ sensors (3 and 6) were more sensitive for the analysis of CPM and PSE in spiked human plasma. The achieved results for the determination of these drugs have demonstrated that a wide concentration range of the drugs could be analyzed by the sensors under investigation with high accuracy and precision as abridged in Table 6.

The response time of the Fe_3_O_4_ sensors was instant (within 5 s), so the sensors were quickly moved between the biological samples and the deionized water in between the measurements in order to prevent the sensing components from adherence on the surface of certain matrix components. It was obvious that the proposed Fe_3_O_4_ sensors could be utilized effectively for in vitro studies. A core benefit of these sensors is their ability to directly determine the target drugs in human plasma samples without the need for any prior treatment or extraction.

As final conclusion, electrodes based on functionalized Fe_3_O_4_ magnetic nanoparticles (sensors 3 and 6) were found to be superior over classical electrodes (sensors 1, 2, 4, and 5) regarding sensitivity (lower LOD), selectivity, response time, and stability.

## 4. Statistical Analysis

Statistical comparison was performed between the six fabricated sensors and a reported method [23] for the analysis of CPM and PSE. As the calculated *t* and *F* values were found to be less than the theoretical values, it was concluded that there was no significant difference as demonstrated in Table 7. One-way ANOVA was also done to ascertain the absence of significant difference between the obtained results of the different proposed sensors (Table 8).

## 5. Conclusion

This work presented a comparative study between potentiometric detection strategy based on functionalized Fe_3_O_4_ magnetic nanoparticles versus classical potentiometric strategy for the quantitative estimation of CPM and PSE. The performance characteristics of the developed sensors were assessed in compliance with the IUPAC recommendations. All the fabricated sensors were satisfactorily simple and selective for the determination of CPM and PSE. The Fe_3_O_4_ sensors have advantages over the membrane sensors of being more sensitive and selective and of having faster response and higher stability. The Fe_3_O_4_ sensors were effectively applied for the analysis of CPM and PSE in both pharmaceutical formulation and spiked human plasma. No interference was observed from coformulated drugs, additives commonly used in dosage form and inorganic substances. The proposed sensors introduced certain advantages as being accurate and do not require drug pretreatment or separation steps which offers a cost-effective method of analysis allowing this method to be used in CPM and PSE routine analysis in quality control laboratories.

## Figures and Tables

**Table 1:** The elemental analysis of ion associates.

Ion-associates	Tentative formulae	Percentage	C%	N%	H%
CPM-PT	[C_16_H_20_ClN_2_]_3_·[PW_12_O_40_]	Found	15.68	2.34	1.70
		Calculated	15.55	2.27	1.62

PSE-PT	[C_10_H_16_NO]_3_·[PW_12_O_40_]	Found	10.83	1.21	1.46
		Calculated	10.66	1.24	1.42

**Table 2:** The composition of the six fabricated sensors.

Sensor	Ion-associate	Plasticizer
1	CPM—PT	DBP
2	CPM—CD	DBP
3	CPM—Fe_3_O_4_ nanoparticles	DBP
4	PSE—PT	DBP
5	PSE—CD	DBP
6	PSE—Fe_3_O_4_ nanoparticles	DBP

**Table 3:** Electrochemical response characteristics of the six investigated membrane sensors.

Parameters	Sensor 1	Sensor 2	Sensor 3	Sensor 4	Sensor 5	Sensor 6
Linearity (mol·L^−1^)	10^−5^–10^−2^	10^−5^–10^−2^	10^−7^–10^−2^	10^−5^–10^−2^	10^−6^–10^−2^	10^−8^–10^−2^
Correlation coefficient	0.9992	0.9994	0.9996	0.9993	0.9994	0.9995
Slope (mV·decade^−1^)^a^	54.6	55.10	58.17	53.70	55.00	57.79
Intercept (mV)^a^	179.6	167.10	316.77	177.2	154.2	274.79
Average recovery (mean ± SD)^a^	100.04 ± 0.886	99.89 ± 0.783	99.97 ± 1.068	100.06 ± 0.897	100.12 ± 1.256	100.01 ± 1.026
LOD (mol·L^−1^)^b^	8 × 10^−6^	9.1 × 10^−6^	5 × 10^−8^	7 × 10^−6^	6.9 × 10^−6^	4 × 10^−9^
Accuracy (mean ± SD)	101.85 ± 1.258	100.66 ± 1.347	100.98 ± 0.813	101.68 ± 0.985	101.64 ± 1.313	99.45 ± 0.973
Precision (RSD %)						
Intraday	1.023	0.998	0.944	1.183	0.994	0.846
Interday	1.155	1.117	0.819	1.063	0.789	0.716
Response time (sec)	10–15	15–20	5–10	10–15	10–15	5–10
Working pH range	4–8	4–8	4–8	4–7	4–7	4–7
Stability (days)	15	20	30	20	25	40

^a^Average of five determinations; ^b^limit of detection (measured by interception of the extrapolated arms of Figure 2).

**Table 4:** Potentiometric selectivity coefficients (*K* ^pot.^) of the six proposed sensors by using the separate solutions method.

Interferent	Sensor 1	Sensor 2	Sensor 3	Sensor 4	Sensor 5	Sensor 6
NaCl	4.51 × 10^−3^	3.67 × 10^−3^	2.14 × 10^−4^	3.17 × 10^−2^	3.26 × 10^−2^	4.57 × 10^−4^
KCl	6.47 × 10^−3^	3.43 × 10^−3^	2.25 × 10^−4^	4.52 × 10^−2^	3.42 × 10^−2^	5.51 × 10^−4^
CaCl_2_	3.22 × 10^−3^	4.27 × 10^−3^	2.67 × 10^−4^	3.63 × 10^−2^	2.68 × 10^−2^	3.17 × 10^−4^
CPM	—	—	—	5.72 × 10^−2^	2.33 × 10^−3^	6.45 × 10^−4^
PSE	3.17 × 10^−1^	3.89 × 10^−2^	8.77 × 10^−3^	—	—	—
IBF	6.83 × 10^−1^	6.51 × 10^−2^	9.25 × 10^−3^	6.83 × 10^−2^	4.47 × 10^−3^	7.48 × 10^−4^

**Table 5:** Determination of CPM and PSE in pharmaceutical formulation by the proposed sensors and comparison with the reported method.

Pharmaceutical formulation		Sensor 3	Sensor 6	Reported method [23]^b^
Sinlerg® tablets labelled to contain 2 mg CPM, 30 mg PSE, and 200 mg IBF/tablet (batch no. 212314)	Mean ± SD^a^	102.26 ± 1.204	101.09 ± 1.106	100.63 ± 1.317
	Student's *t* test	0.518 (2.26^b^)	0.743 (2.26^b^)	
	*F* test	1.623 (6.26^b^)	1.182 (6.26^b^)	

^a^Average of five determinations. ^b^Reported HPLC method using C18 column; flow rate 1.5 mL·min^−1^; mobile phase is a gradient elution of acetonitrile:buffer (15 : 85, v/v) for 5.5 min, (45 : 55, v/v) for 5.5–12 min, and (60 : 40, v/v) for 12–17 min at pH = 3 and UV detection at 220 nm.

**Table 6:** Determination of CPM and PSE in spiked human plasma by the proposed sensors 3 and 6.

Drug concentration	Spiked human plasma	
	Sensor 3	Sensor 6
	Recovery (%) ± SD^*∗*^	
10^−5^ M	101.12 ± 0.716	100.41 ± 1.215
10^−6^ M	100.60 ± 1.158	100.31 ± 1.040
10^−7^ M	101.78 ± 0.618	101.39 ± 0.622

^*∗*^The recovery percentages are the average of three determinations.

**Table 7:** Statistical comparison between the results obtained by the proposed sensors and the reported method for the determination of CPM and PSE in pure form.

Parameter		Mean^a^	SD	Variance	*n*	*t* test	*F* test
CPM	Sensor 1	100.04	0.886	0.785	4	0.484 (2.36^b^)	1.445 (9.12^b^)
	Sensor 2	99.89	0.783	0.613	4	0.265 (2.36^b^)	1.850 (9.12^b^)
	Sensor 3	99.97	1.068	1.141	6	0.384 (2.26^b^)	1.006 (6.26^b^)
	Reported method [23]^c^	99.72	1.065	1.134	5		

PSE	Sensor 4	100.06	0.897	0.804	4	0.943 (2.36^b^)	1.271 (9.12^b^)
	Sensor 5	100.12	1.256	1.577	5	0.944 (2.31^b^)	1.543 (6.39^b^)
	Sensor 6	100.01	1.026	1.052	7	0.951 (2.23^b^)	1.029 (6.16^b^)
	Reported method [23]^c^	99.45	1.011	1.022	5		

^a^Average of six determinations; ^b^figures between parentheses represent the corresponding tabulated values of *t* and *F* at *P*=0.05; ^c^reported HPLC method using C18 column, flow rate 1.5 mL·min^−1^, mobile phase is a gradient elution of acetonitrile:phosphate buffer (15 : 85, v/v) for 5.5 min, (45 : 55, v/v) for 5.5–12 min, and (60 : 40, v/v) for 12–17 min at pH = 3 and UV detection at 220 nm.

**Table 8:** One-way ANOVA testing for the different proposed methods and the reported method used for the determination of CPM and PSE.

	Source	Sum of squares	Degree of freedom^a^	Mean squares	*F* value^b^	*P* value	*F* critical
PSE	Between experiment	0.2618	3	0.0873	0.0750	0.9726	3.1968
	Within experiment	19.7883	17	1.1640			

CPM	Between experiment	0.1858	3	0.0619	0.0609	0.9796	3.2874
	Within experiment	15.2688	15	1.0179			

^a^At the 0.05 level; ^b^the population means are not significantly different.

## Data Availability

All the data are included in the manuscript in the form of provided tables and figures.
